# Supplementary material for: In-Depth Immunophenotyping With Mass Cytometry During TB Treatment Reveals New T-Cell Subsets Associated With Culture Conversion
Source: Front Immunol. 2022 Mar 22;13:853572. doi: 10.3389/fimmu.2022.853572 (PMC8980213; doi:10.3389/fimmu.2022.853572)
Supplement: Supplementary file 1 [file DataSheet_1.docx]

# Supplementary Figures


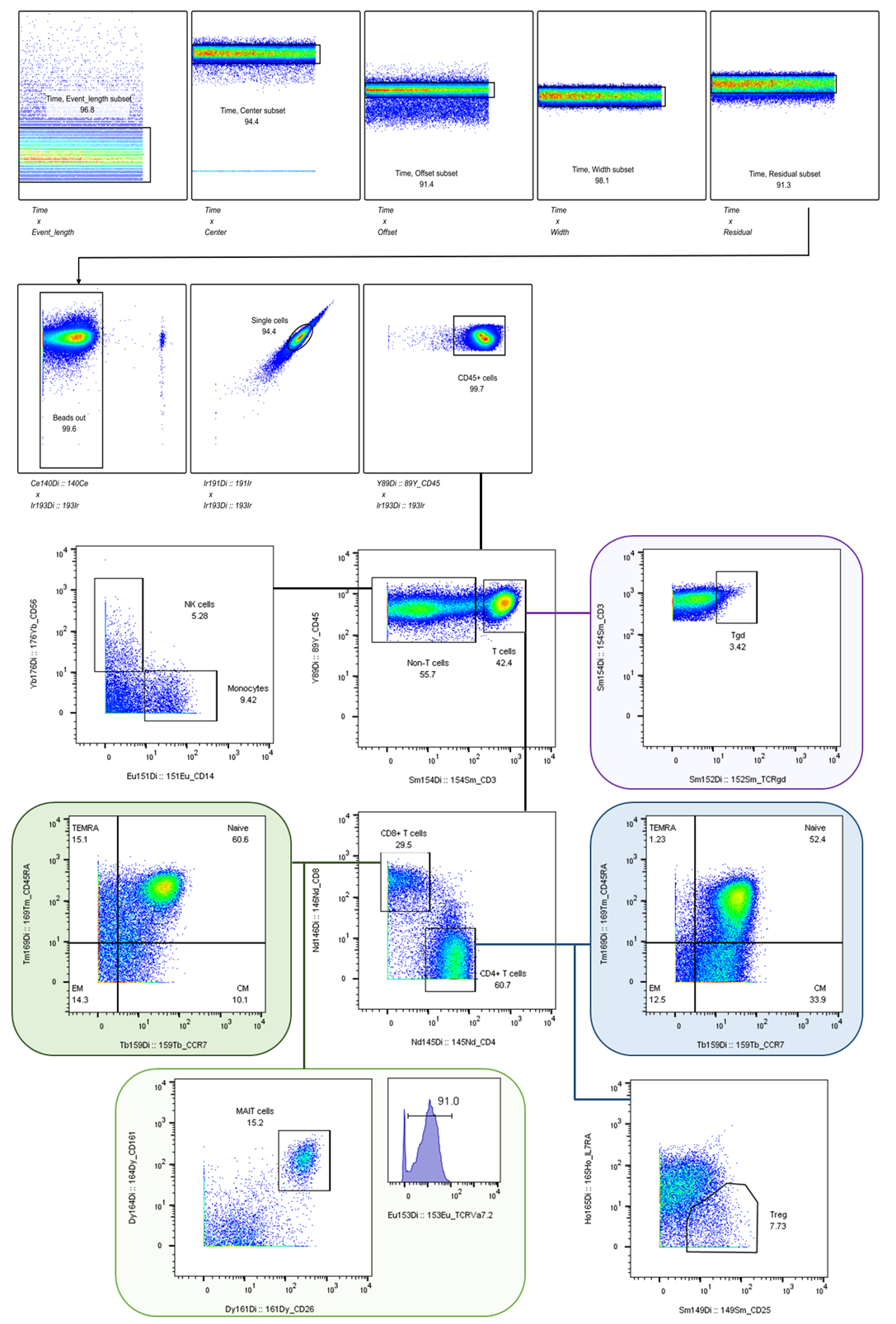


**Supp. Figure 1. Main CD45^+^ non-granulocyte whole blood subpopulations and T-cell oriented gating strategy.** CM: central memory. EM: effector memory MAIT: mucosal-associated invariant T-cells. NK: natural killers. TEMRA: terminally differentiated effectors re-expressing CD45RA. Tgd: gamma delta T-cells. Treg: T regulators.


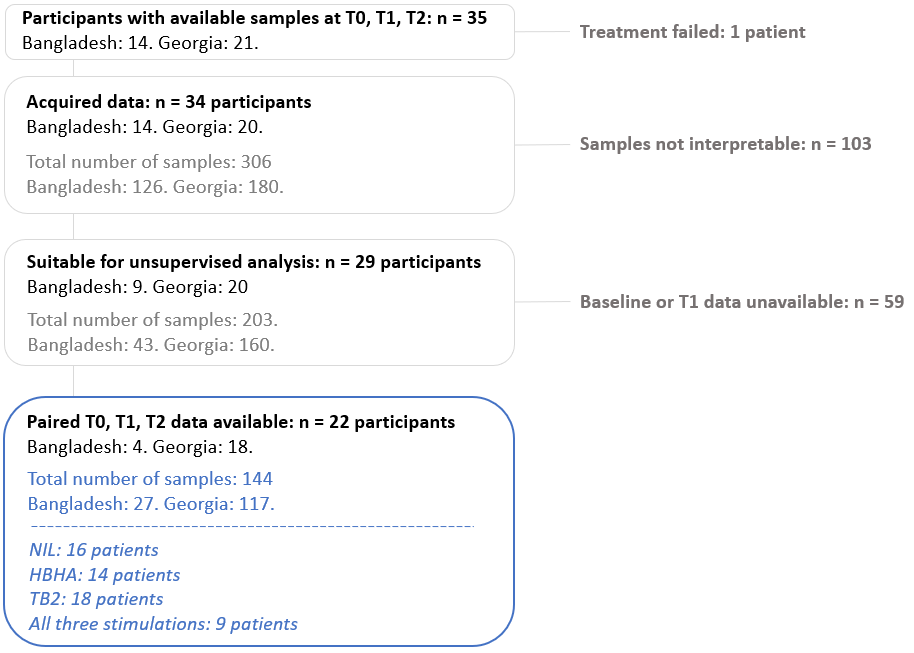


**Supp. Figure 2. Flowchart of patient inclusions.**

A table listing the number of cell samples available for each stimulation condition and each patient individually is provided in Supp Table 1.


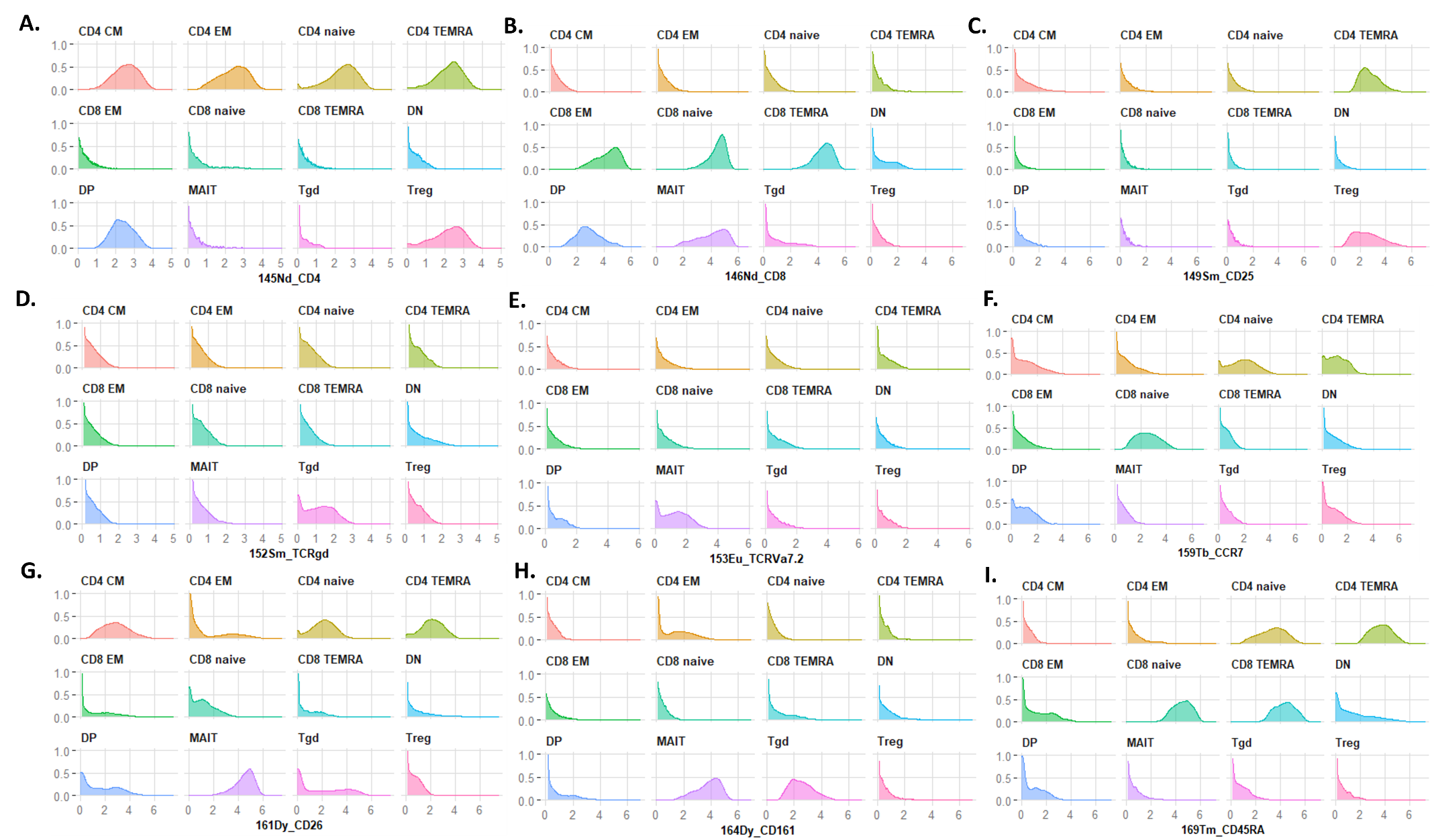


**Supp. Figure. 3. Histogram density plot of marker expression in the 12 cell populations identified by FlowSOM.** Data are shown for the nine markers which were used as clustering channels (see Supp. Table 3). **A.** CD4. **B.** CD8. **C.** CD25. **D.** TCRgd. **E.** TCRVa7.2. **F.** CCR7. **G.** CD26. **H.** CD161. **I.**  CD45RA.

**
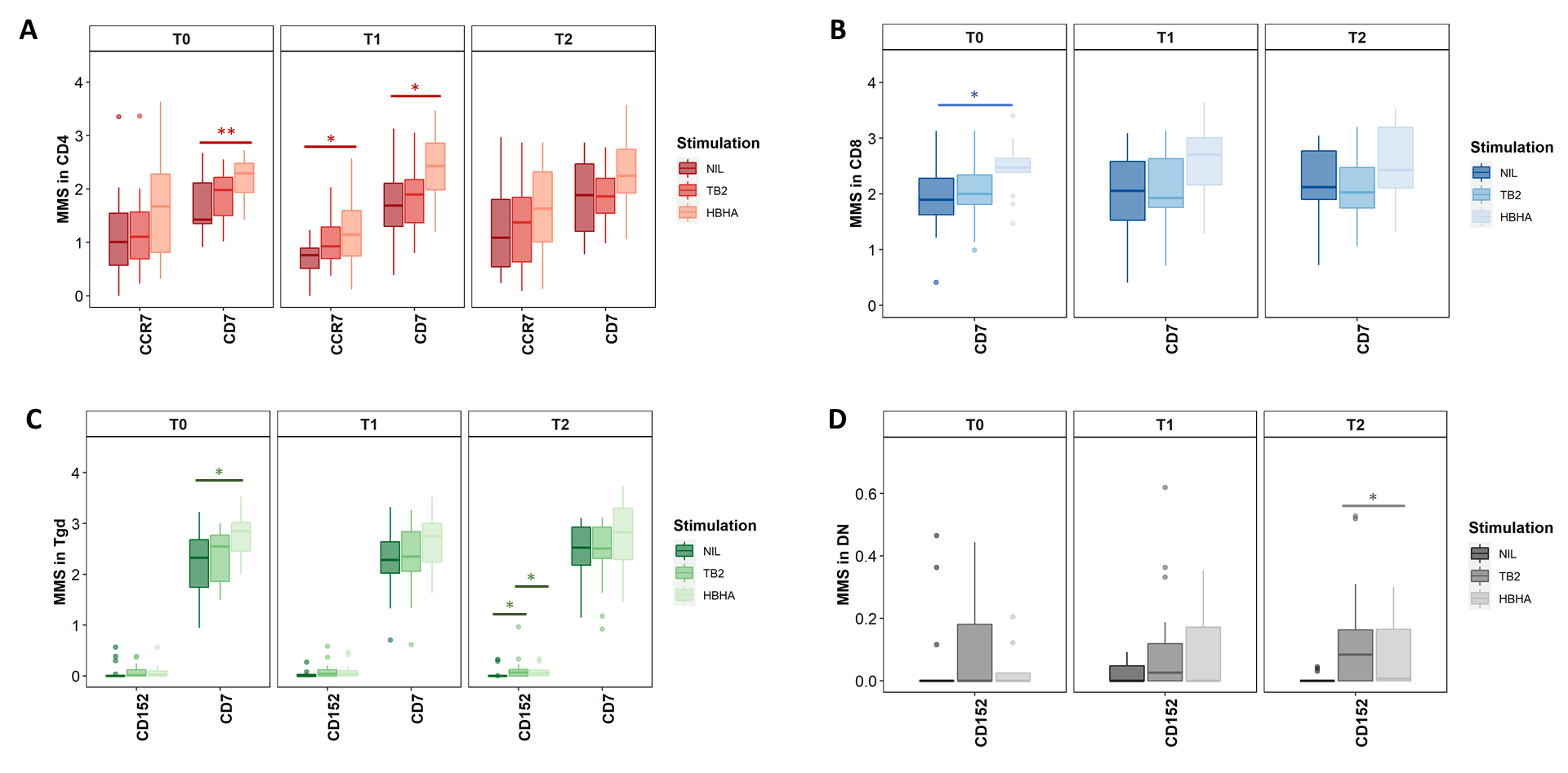
**

**Supp. Figure. 4. Impact of *in vitro* whole blood stimulation with *Mtb* antigens on surface marker expression in the main T-cell subpopulations.**

The surface expression of all panel markers was compared between the three stimulation conditions (unstimulated (NIL), TB2, and rmsHBHA) in CD4^+^ (**A**), CD8^+^ (**B**), gamma-delta (Tgd; **C**), or double negative (DN) T-cells (**D**). MMS: median mass signal. Only the markers for which a significant difference was observed were represented. Statistical analysis: two-sided Kruskal-Wallis test with Dunn’s Kruskal–Wallis Multiple Comparisons post-hoc at T0, T1, and T2. *: p<0.05. **: p<0.01. Number of data points per timepoint for all panels: NIL: n = 16. TB2: n = 18. HBHA: n = 14. Exact p-values and test statistics are available in Supp. Table 4.

**
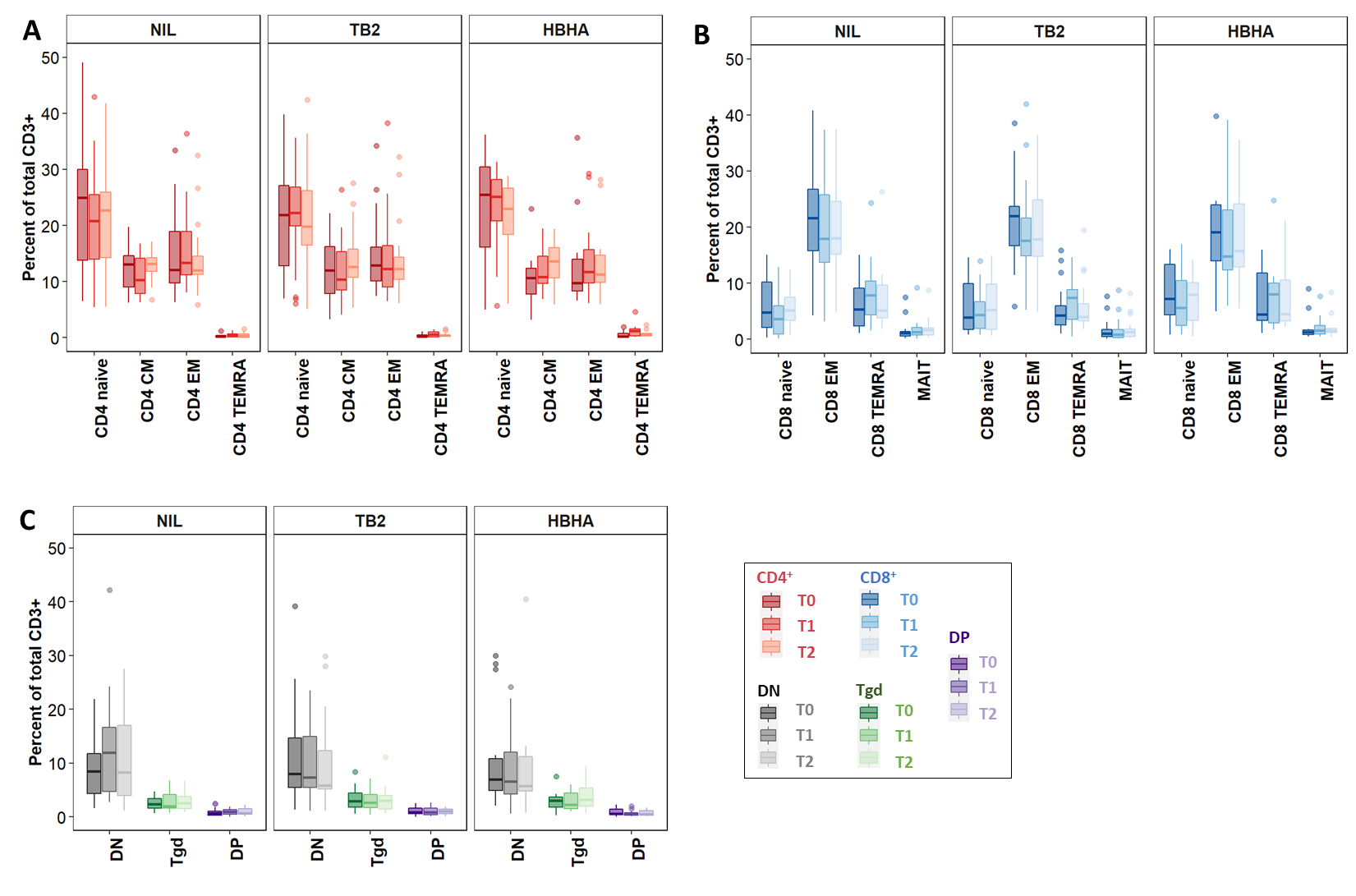
**

**Supp. Figure. 5. Frequencies of the main peripheral T-cell subpopulations throughout anti-TB treatment.**

Evolution of the frequency of canonical T-cell subsets identified through FlowSOM meta-clustering and corresponding respectively to CD4^+^ phenotypes (**A**), CD8^+^ phenotypes (**B**), or other cell subsets (**C**). Number of data points per timepoint for all panels: NIL: n = 16. TB2: n = 18. HBHA: n = 14. Data are given as median + interquartile range. Abbreviations: CM: central memory. DN: double-negative CD4^-^CD8^-^. DP: double-positive CD4^+^ CD8^+^. EM: effector memory. HBHA: recombinant *M. tuberculosis* heparin-binding hemagglutinin. MAIT: mucosal associated invariant T-cells. NIL: unstained control. TB2: *M. tuberculosis* antigenic peptide pool. Tgd: gamma delta T-cells. Treg: T-regulators. TEMRA: terminally differentiated effectors re-expressing CD45RA. No statistically significant differences were detected (pairwise comparisons between non-sindependent observations at T0, T1, and T2: two-sided Friedman rank sum test and Wilcoxon-Nemenyi-Thompson post-hoc for pairwise comparisons between non-independent observations at T0, T1, and T2).

**
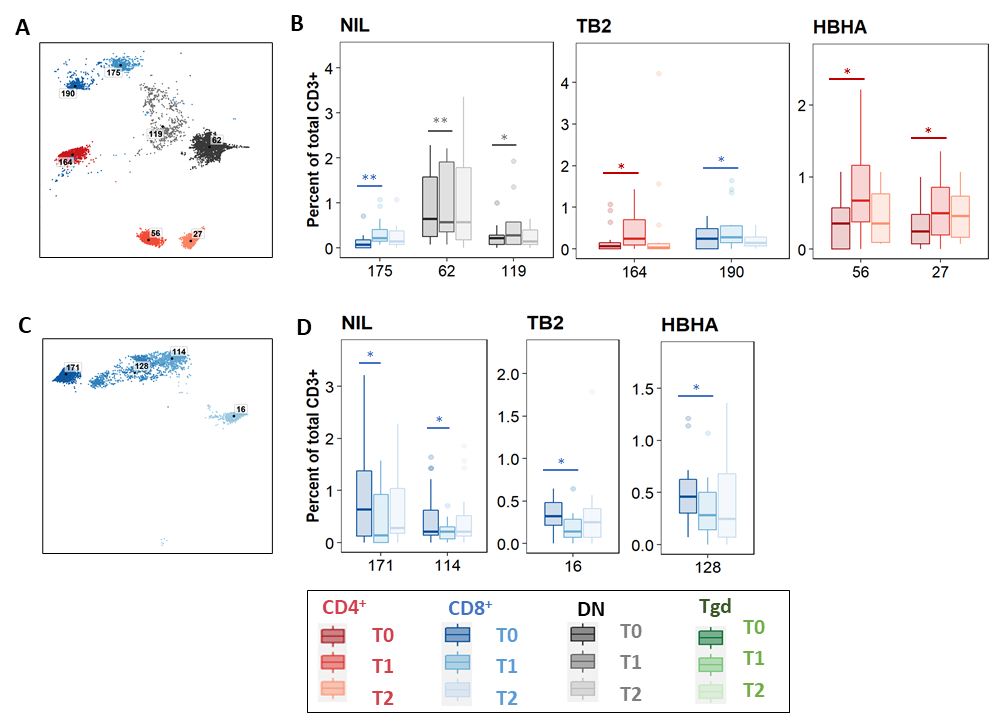
**

**Supp Figure 6. Significant abundance changes in non-canonical T-cell subsets after the intensive phase of treatment.** The evolution of FlowSOM cluster abundance was analyzed over time in unstimulated or *Mtb*-stimulated samples (TB2 or rmsHBHA), and only the clusters within which significant abundance changes were detected were displayed. CD4^+^ clusters were represented in red, CD8^+^ clusters in blue, γδ T-cell clusters in green, and CD4^-^ CD8^-^ clusters in grey. Number of matched data points per timepoint for all panels: NIL: n = 16. TB2: n = 18. rmsHBHA: n = 14. Data are given as median + interquartile range.

**A and B.** Significantly increased clusters at the end of the intensive phase of treatment (T1) compared to treatment initiation (T0). Clusters within which a significant increase was detected between T0 and T1 were first visualized on the reference UMAP (**A**). Cluster abundance quantification was then was performed in unstimulated, TB2-stimulated or rmsHBHA-stimulated samples (**B**).

**C and D**. Significantly decreased clusters at the end of the intensive phase of treatment (T1) compared to treatment initiation (T0). Mapping **(C**) and abundance quantification of clusters which decreased between T0 and T1 in unstimulated, TB2-stimulated, or rmsHBHA-stimulated samples (**D**).

Statistical analysis: two-sided Friedman rank sum test and Wilcoxon-Nemenyi-Thompson post-hoc for pairwise comparisons between non-independent observations at T0, T1, and T2. *: p<0.05. **: p<0.01. ***: p<0.001. Exact p-values and test statistics are available in Supp. Table 5. Benjamini-Hochberg corrections for multiple comparisons were performed as an indication and were not used for cluster selection for phenotype analyses in order to minimize type II errors. Adjusted p-values did not reach significance. All adjusted p-values and complete test statistics are available in Supp. Table 5.

**
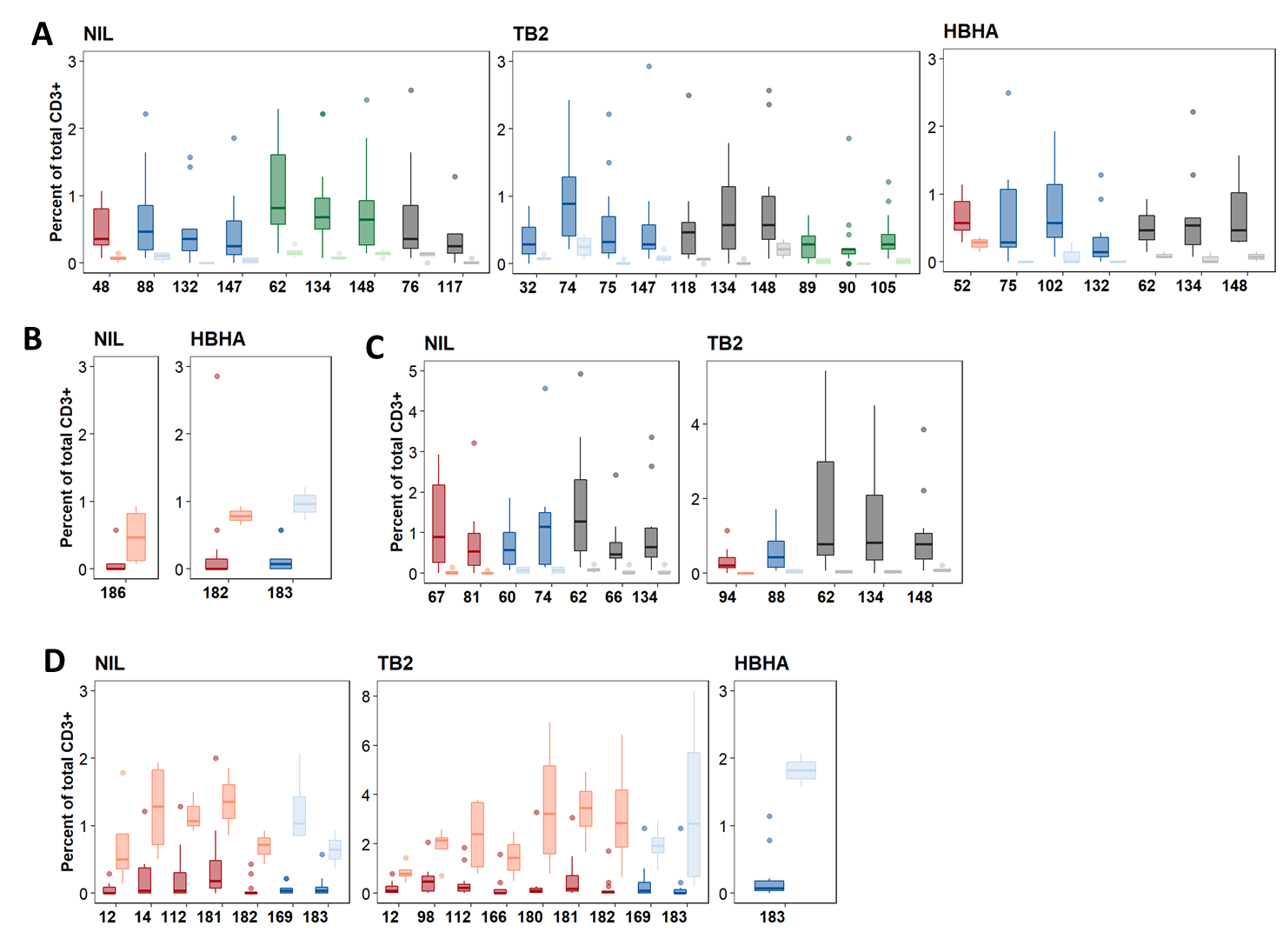
**

**Supp. Figure. 7. Patients with slow microbiological culture conversion show decreased CD8^+^ and γδ and enriched CD4^+^ naïve peripheral T-cell subsets during treatment.**

Clusters with differential abundance between patients with positive mycobacterial cultures at T1 (slow converters, n = 4) or with negative cultures at T1 (fast converters, n = 18). Data are shown as median + interquartile range.

**A and B.** **At treatment initiation (T0).** Clusters significantly decreased (**A**) and increased (**B**) in slow converters compared to fast converters.

**C and D. At treatment completion (T2).** Clusters significantly decreased (**C**) and increased (**D**) in slow converters compared to fast converters.

CD4^+^ clusters are represented in red, CD8^+^ clusters in blue, Tgd clusters in green, and DN clusters in grey. The lighter shade of each color code corresponds to data from the slow converters. Statistical analysis: two-sided Mann-Whitney U-test. For all represented clusters: P<0.025 at T0; P<0.013 at T2. Significance stars were not displayed for readability. Benjamini-Hochberg corrections for multiple comparisons were performed as an indication and were not used for cluster selection for phenotype analyses in order to minimize type II errors. Adjusted p-values did not reach significance. All exact and adjusted p-values as well as complete test statistics are available in Supp. Table 8.

**
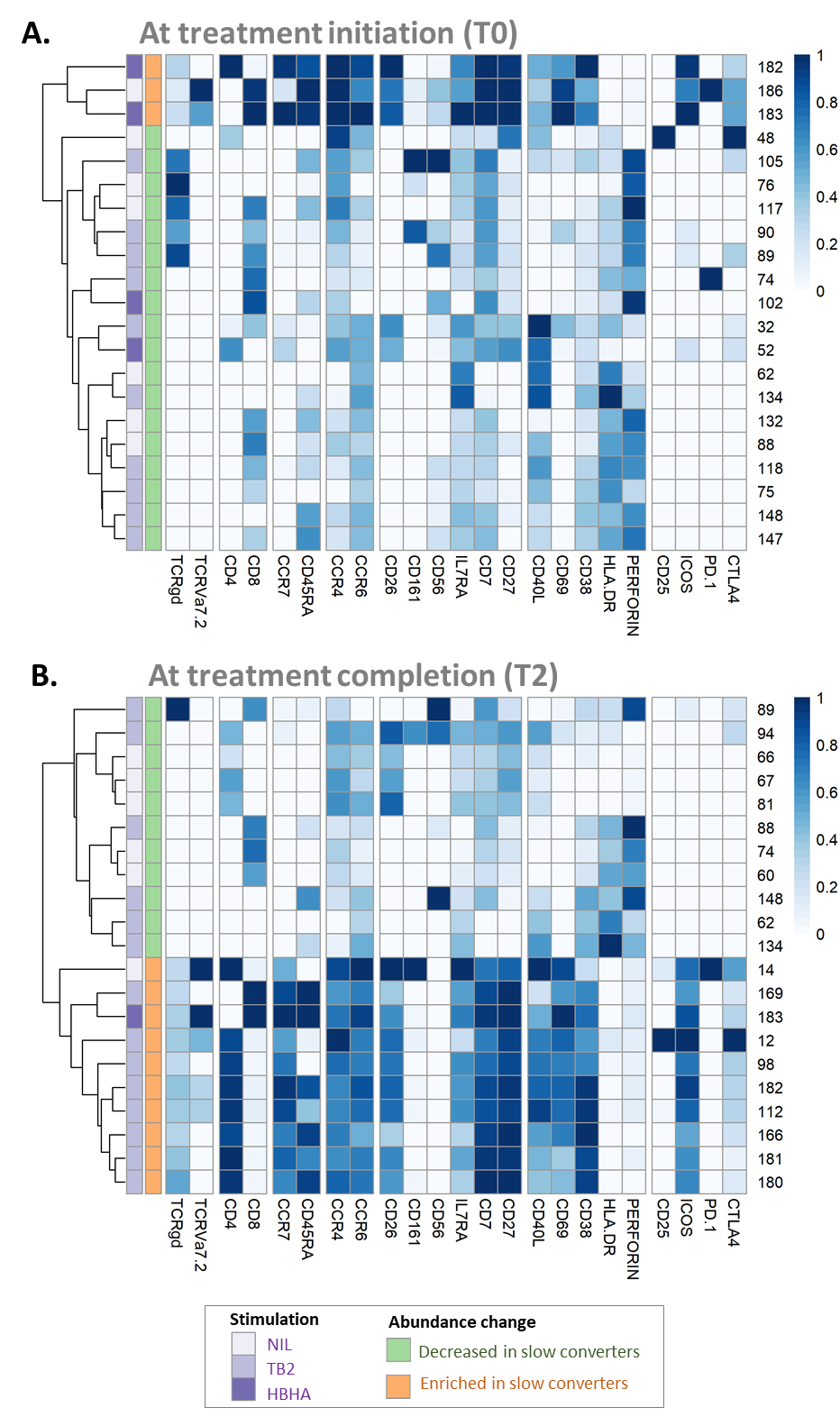
**

**Supp. Figure 8.** **Patients with slow microbiological culture conversion show decreased cytotoxic CD8^+^ and γδ and enriched CD4^+^ naïve T-cell subsets before treatment initiation and after treatment completion compared to fast converters.** Fast converters (n = 18) were defined as patients with permanently negative *M. tuberculosis* culture after the intensive phase of treatment (T1), whereas slow converters (n = 4) were defined as patients with persistently positive cultures at T1. The abundance of all FlowSOM clusters at baseline was compared between fast and slow converters. Only clusters within which significant differences were detected were represented (T0: p<0.026. T2: p<0.013; two-sided Mann-Whitney U test; unadjusted p-values, see Supp. Figure 5).

**A. Before treatment initiation (T0).** Clusters which were significantly decreased (green) or increased (orange) at T0 in slow converters compared to fast converters were represented. Normalized, arcsinh-transformed mean marker expression levels were visualized). Each line represents one cluster. Scales indicate normalized mass signal intensity.

**B. After treatment completion (T2).** Clusters which were significantly increased or decreased at T2 in slow converters compared to fast converters were represented and marker expression levels were visualized. All patients achieved microbiological cure at T2.


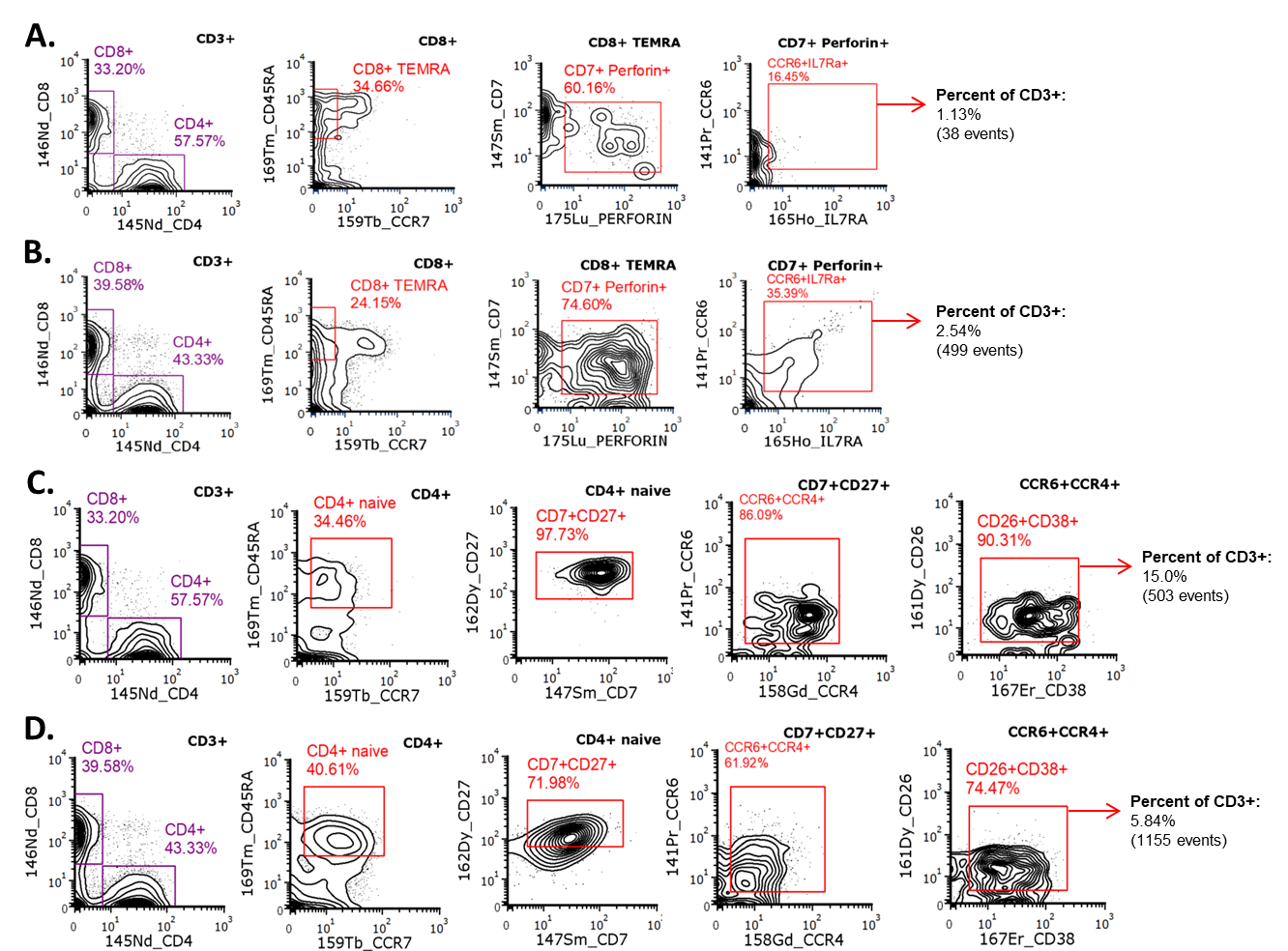


**Supp. Figure 9.** **Example of manual gating validation of clusters identified in Figure 6.**

Two cell subpopulations representative of the main cluster subsets identified in Figure 6 were gated manually, and cell frequencies were compared at T1 between TB2 stimulated samples from a patient with a positive sputum culture (slow converter, **A.** and **C.**) and patient with a negative sputum culture (fast converter, **B.** and **D**) using the same gates.

Phenotypes indicated in bold above biaxial plots represent the parent population.

**A. and B. Cytotoxic CD8^+^ T-cells.** Target phenotype CD3^+^CD8^+^CCR7^-^CD45RA^+^CD27^+^Perforin^+^CCR6^+^IL7RA^+^. In Figure 6, this corresponds to the phenotype of clusters 146, 132, 148, 119, 147, 116.

**C. and D. Naïve CD4^+^ T-cells** Target phenotype: CD3^+^CD4^+^CCR7^+^CD45RA^+^CD7^+^CD27^+^CCR6^+^CCR4^+^CD26^+^CD38^+^. In Figure 6, this corresponds to the phenotype of clusters 125, 178, 180, 166.

**Supplementary Table 1. Sociodemographic and clinical characteristics of the cohort.**

|  | **Nb of cell samples** | | | **Drug susceptibility** | | |
| --- | --- | --- | --- | --- | --- | --- |
| **ID** | **NIL** | **TB2** | **HBHA** | **Phenotypic drug susceptibility** | **Treatment regimen** | **Country** |
| 01DSGE | 3 | 3 | 3 | DS | 2HRZE/4HRE | GEO |
| 02DSGE | 3 | 0 | 3 | DS | 2HRZE/4HRE | GEO |
| 03DSGE | 3 | 3 | 3 | DS | 2HRZE/4HR | GEO |
| 04DSGE | 3 | 0 | 0 | DS | 2HRZE/4HR | GEO |
| 05DSGE | 3 | 3 | 3 | DS | 2HRZE/4HR | GEO |
| 06DSB | 3 | 3 | 0 | DS | 2HRZE/4HR | BD |
| 06DSGE | 3 | 3 | 0 | DS | 2HRZE/4HRE | GEO |
| 07DSGE | 3 | 3 | 3 | DS | 2HRZE/4HRE | GEO |
| 08DSB | 3 | 3 | 0 | DS | 2HRZE/4HR | BD |
| 08DSGE | 3 | 3 | 3 | DS | 2HRZE/4HRE | GEO |
| 21DSB | 0 | 3 | 3 | DS | 2HRZE/4HR | BD |
| 01DRGE | 0 | 3 | 0 | MDR | H+Z+Km+Lfx+cfz+Bdq / Z+Lfx+cfz+Bdq | GEO |
| 02DRGE | 3 | 3 | 3 | MDR | Cm+Lfx+Pto+Cs+Cfz+H / Cm+Lfx+Cs+Bdq+Lzd / Lfx+Cs+Bdq+Lzd | GEO |
| 03DRGE | 3 | 0 | 3 | Pre-XDR | (Z)+Lfx+Pto+Cfz+Bdq+Lzd / Cs+Cfz+Bdq+Lzd | GEO |
| 04DRGE | 0 | 3 | 0 | Pre-XDR | Bdq+Cfz+Lfx+H+Pto+E+Z / Bdq+cfz+Lzd+H+E / Bdq+Cfz+Lfx+H+Z / Bdq+Cfz+Lfx+E+Z | GEO |
| 05DRGE | 3 | 3 | 0 | Pre-XDR | H+Z+Km+Lfx+Cfz+Bdq / Z+Lfx+Cfz+Bdq | GEO |
| 06DRGE | 0 | 3 | 3 | RR | H+E+Z+Km+Mfx+Pto+Cfz / Mfx+E+Z+cfz | GEO |
| 07DRGE | 3 | 3 | 3 | XDR | Bdq+Lzd+cfz+Cs | GEO |
| 09DRB | 3 | 3 | 3 | MDR | 4-6 Km-Mfx-Pto-Cfz-Z-HHigh dose-E/5-6 Mfx-Cfz-Z-E3 | BD |
| 09DRGE | 0 | 0 | 3 | MDR | H+Z+Km+Lfx+Cfz+Bdq / Z+Lfx+Cfz+Bdq | GEO |
| 10DRGE | 0 | 3 | 0 | MDR | H+Z+Km+Lfx+Cfz+Bdq / Z+Lfx+Cfz+Bdq | GEO |
| 14DRGE | 3 | 3 | 3 | Pre-XDR | H+Z+Km+Lfx+Cfz+Bdq / Z+Lfx+Cfz+Bdq | GEO |
| ***Nb of patients:*** | *16* | *18* | *14* |  |  |  |
| **Number of samples:** | 48 | 54 | 42 |  |  |  |
| **Total number of samples:** | **144** | | |  |  |  |

Footnotes: Three samples per patient were collected in each stimulation condition, corresponding to each timepoint: T0: baseline. T1: T0 + 2 months. T2: end of treatment.Samples with < 1,000 CD3^+^ events, and batches with missing samples from a given timepoint were removed from the analysis. Abbreviations: BD: Bangladesh. GEO: Georgia. DS: drug susceptible. MDR: multi-drug resistant. RR: rifampicin resistant. XDR: extensively drug resistant. Abbreviations for anti-TB drugs: E: Ethambutol. H: Isoniazid. R: Rifampicin. S: Streptomycin. Z: Pyrazinamide. Bdq: Bedaquiline. Cfz: Clofazimine. Cs: Cycloserine. Km: Kanamycin. Lfx: Levofloxacin. Lzd: Linezolid. Mfx: Moxifloxacin. Pto: Prothionamide. Numbers indicate months of treatment when the information was available.

**Supplementary Table 1 - continued**

| **Baseline identification** | | | | | | | | | | | **Microbiological evolution** | | | | | | | |
| --- | --- | --- | --- | --- | --- | --- | --- | --- | --- | --- | --- | --- | --- | --- | --- | --- | --- | --- |
| **ID** | **Gender** | **Age** | **Occupation** | **HIV** | **Diabetes** | **BCG** | **QFT-P** | **GeneXpert** | **GeneXpert RIFR** | ***Mtb* culture** | | | | **AFB** | | | **Treatment response group** |  |
|  |  |  |  |  |  |  |  |  |  | **T0** | | **T1** | **T2** | **T0** | **T1** | **T2** |  |  |
| 01DSGE | M | 28 | Small business | n | n |  | + | + | - | + | | - | - | scanty | scanty | - | Fast |  |
| 02DSGE | M | 26 | Student | n | n | y | + | + | - | + | | - | - | - | - | - | Fast |  |
| 03DSGE | M | 37 | Small business | n | n | y | + | + | - | + | | - | - | - | - | - | Fast |  |
| 04DSGE | M | 24 | Day Laborer | n | n | y | + | + | - | + | | - | - | - | - | - | Fast |  |
| 05DSGE | M | 63 | Unemployed / Retired | n | n |  | + | + | - | + | | - | - | 2+ | 2+ | - | Fast |  |
| 06DSB | M | 16 | Day Labour | n | n | y | + | + | - | + | | - | - | 2+ | scanty | - | Fast |  |
| 06DSGE | M | 58 | Unemployed / Retired | n | n |  | - | + | - | + | | + | - | 1+ | 1+ | scanty | Slow |  |
| 07DSGE | M | 50 | Unemployed / Retired | n | n |  | + | + | - | + | | + | - | scanty | scanty | - | Slow |  |
| 08DSB | M | 60 | Small business | n | n | y | - | + | - | + | | - | - | 1+ | - | - | Fast |  |
| 08DSGE | F | 29 | Small business | n | n | y | + | + | - | + | | + | - | scanty | scanty | - | Slow |  |
| 21DSB | F | 20 | Student | n | n | y | + | + | - | + | | - | - | 2+ | - | - | Fast |  |
| 01DRGE | M | 42 | Farmer | n | n |  | - | + | - | + | | - | - | 1+ | - | - | Fast |  |
| 02DRGE | F | 34 | Housewife | n | n | y | - | + | + | + | | + | - | - | - | - | Slow |  |
| 03DRGE | M | 26 | Business | n | n | y | + | + | - | + | | - | - | 2+ | - | - | Fast |  |
| 04DRGE | M | 37 | Private Service | n | n |  | + | + | - | + | | - | - | - | 1+ | - | Fast |  |
| 05DRGE | M | 42 | Unemployed | n | n |  | + | + | - | + | | - | - | 3+ | scanty | - | Fast |  |
| 06DRGE | F | 33 | Unemployed | n | n |  | + | + | + | + | | - | - | 2+ | - | - | Fast |  |
| 07DRGE | F | 20 |  | n | n | y | - | + | + | + | | - | - | 2+ | - | - | Fast |  |
| 09DRB | F | 15 | Garments worker | n | n | y | - | + | + | + | | - | - | 3+ | - | - | Fast |  |
| 09DRGE | F | 28 |  | n | n |  | + | + | - | + | | - | - | 2+ | - | - | Fast |  |
| 10DRGE | F | 25 |  | n | n |  | - | + | + | + | | - | - | - | - | - | Fast |  |
| 14DRGE | M | 31 |  | n | n |  | + | + | + | + | | - | - | scanty | scanty | scanty | Fast |  |

Footnotes**:** BCG: Bacille Calmette-Guérin vaccination. QFT-P: QuantiFERON-TB Gold Plus. AFB: Acid Fast Bacilli detection (sputum smear microscopy). BMI: body mass index. *Mtb: Mycobacterium tuberculosis*. Throughout the table, "+" and "-" indicate positive or negative results to the indicated test. For AFB results, 1+, 2+, or 3+ quantify the amount of bacilli observed.

**Supplementary Table 1 - continued**

| **Weight evolution** | | | | | | | **TB risk factors** | | | | | |
| --- | --- | --- | --- | --- | --- | --- | --- | --- | --- | --- | --- | --- |
| **ID** | **Weight (kg)** | | | **BMI** | | | **Smoking** | **Alcohol** | **Intraveinous drug use** | **Prison** | **TB contact** | **Previous TB** |
|  | **T0** | **T1** | **T2** | **T0** | **T1** | **T2** |  |  |  |  |  |  |
| 01DSGE | 72 | 72 | 73 | 20.8 | 20.8 | 21.1 | n | n | n | n | n | n |
| 02DSGE | 59 | 61 | 64 | 20.1 | 20.8 | 21.8 | n | n | n | n | n | n |
| 03DSGE | 65 | 66 | 71 | 24.1 | 24.5 | 26.3 | y | n | n | n | n | n |
| 04DSGE | 59 | 60 | 64 | 19.9 | 20.2 | 21.6 | y | n | n | n | n | n |
| 05DSGE | 66.7 | 67 | 68 | 24.2 | 24.3 | 24.6 | n | n | n | n | n | n |
| 06DSB | 42.2 | 43.3 | 48.1 | 16.4 | 16.9 | 18.7 | n | n | y | n | n | n |
| 06DSGE | 64.2 |  |  | 18.3 |  |  | y |  | n | n | n | n |
| 07DSGE | 64 | 64 | 67 | 19.7 | 19.7 | 20.6 | y | n | n | n | n | n |
| 08DSB | 52.5 | 57.5 | 59 | 18.1 | 19.8 | 20.4 | y | n | n | n | n | n |
| 08DSGE | 60 | 61 | 67 | 24.3 | 24.7 | 27.1 | n | n | n | n | n | y |
| 21DSB | 36.7 | 37 | 44.5 | 13.3 | 13.4 | 16.1 | n | n | n | n | n | n |
| 01DRGE | 66 | 66 | 69 | 23.9 | 23.9 | 25.0 | y | y | n | n | n | n |
| 02DRGE | 48 | 52 | 54 | 18.7 | 20.3 | 21.0 | n | n | n | n | n | n |
| 03DRGE | 54 | 58 | 71 | 17.2 | 18.5 | 22.6 | y | n | n | n | n |  |
| 04DRGE | 71 | 72 | 73 | 20.5 | 20.8 | 21.1 | y | n |  | n | n | n |
| 05DRGE | 60 | 62 | 65 | 19.8 | 20.4 | 21.4 | y | n | n | n | n | y |
| 06DRGE | 48 | 52 | 56 | 18.7 | 20.3 | 21.8 | n | n | n | n | n | n |
| 07DRGE | 54 | 57 | 61 | 19.5 | 20.6 | 22.1 | n | n | n | n | n | n |
| 09DRB | 43.2 | 45.5 | 44.9 | 17.9 | 18.9 | 18.6 | n | n | n | n | y | y |
| 09DRGE |  | 60 | 60 |  |  |  | n | n | n | n | n | n |
| 10DRGE | 59 | 59 | 63 | 19.9 | 19.9 | 21.2 | n | n | n | n |  | n |
| 14DRGE | 60 | 62 | 66 | 20.7 | 21.4 | 22.8 | y | n | n | n | y | n |

Footnotes: BMI: body mass index.

**Supplementary Table 2. Mass cytometry panel components.**

| **Cellular target** | **Clone** | **Metal tag** | **Volume for 10^6^ cells**  **(in 100µL total)** |
| --- | --- | --- | --- |
| CD45 | HI30 | 89Y | 1,5 |
| CD196/CCR6 | 11A9 | 141Pr | 0,5 |
| CD69 | FN50 | 144Nd | 1 |
| CD4 | RPA-T4 | 145Nd | 1 |
| CD8 | RPA-T8 | 146Nd | 0,7 |
| CD7 | CD7-6B | 147Sm | 1 |
| CD278/ICOS | C398.4A | 148Nd | 0,7 |
| CD25 | 2A3 | 149Sm | 1 |
| CD14 | RMO52 | 151Eu | 1,5 |
| TCRgd | 11F2 | 152Sm | 1 |
| TCRVa 7.2 | 3C10 | 153Eu | 1,5 |
| CD3 | UCHT1 | 154Sm | 1 |
| CD279/PD-1 | EH12.2H7 | 155Gd | 1 |
| CD183/CXCR3 | G025H7 | 156Gd | 1 |
| CD194/CCR4 | L291H4 | 158Gd | 1 |
| CD197/CCR7 | G043H7 | 159Tb | 1 |
| CD26 | BA5b | 161Dy | 1 |
| CD27 | L128 | 162Dy | 1 |
| CD161 | HP-3G10 | 164Dy | 1 |
| CD127/IL-7Ra | A019D5 | 165Ho | 0,7 |
| CD38 | HIT2 | 167Er | 1 |
| CD154/CD40L | 24-31 | 168Er | 1 |
| CD45RA | HI100 | 169Tm | 1 |
| CD152/CTLA-4 | 14D3 | 170Er | 1 |
| CD185/CXCR5 | RF8B2 | 171Yb | 1 |
| HLA-DR | L243 | 173Yb | 0,7 |
| Perforin | B-D48 | 175Lu | 1 |
| CD56 | NCAM16.2 | 176Yb | 1 |
| CD16 | 3G8 | 209Bi | 1 |

Footnotes: all antibodies were supplied by Fluidigm.

**Supplementary Table 3. Definition of clustering channels and expected cell subpopulations for dimension reduction and automated clustering of CD3^+^ T-cells.**

| **Clustering channels** | **Expected cell subpopulations** | **Phenotype** | **Average % with manual gating^1^** | **Yielded % with FlowSOM metaclustering** |
| --- | --- | --- | --- | --- |
| CD4  CD8  CCR7  CD45RA  CD161  CD26  TCRgd TCRVa7.2  CD25 | Gamma delta T-cells | CD3^+^ TCRγδ^+^ | 3.3 | 2.86 |
|  | MAIT-cells | CD3^+^ CD4^-^ CD8^+^ CD26^+^ CD161^+^ TCRVα7.2^+^ | 2.2 | 1.57 |
|  | Naive CD8^+^ T-cells | CD3^+^ CD4^-^ CD8^+^ CCR7^+^ CD45RA^+^ | 12.6 | 7.3 |
|  | Effector memory CD8^+^ T-cells | CD3^+^ CD4^-^ CD8^+^ CCR7^-^ CD45RA^-^ | 8.0 | 13.9 |
|  | Central memory CD8^+^ T-cells | CD3^+^ CD4^-^ CD8^+^ CCR7^+^ CD45RA^-^ | 0.9 | *Not detected* |
|  | TEMRA CD8^+^ T-cells | CD3^+^ CD4^-^ CD8^+^ CCR7^-^ CD45RA^+^ | 9.7 | 10.7 |
|  | Naive CD4^+^ T-cells | CD3^+^ CD4^+^ CD8^-^ CCR7^+^CD45RA^+^ | 20.2 | 13.9 |
|  | Effector memory CD4^+^ T-cells | CD3^+^ CD4^+^ CD8^-^ CCR7^-^CD45RA^-^ | 12.6 | 29.7 |
|  | Central memory CD4^+^ T-cells | CD3^+^ CD4^+^ CD8^-^ CCR7^+^CD45RA^-^ | 9.7 | 6.0 |
|  | TEMRA CD4^+^ T-cells | CD3^+^ CD4^+^ CD8^-^ CCR7^-^ CD45RA^+^ | 2.3 | 1.0 |
|  | Treg | CD3^+^ CD4^+^ CD25^+^ IL7Ra^-^ | 2.1 | 1.7 |
|  | Double negative T-cells | CD3^+^ CD4^-^ CD8^-^ | 7.4 | 2.4 |
|  | Double positive T-cells | CD3^+^ CD4^+^ CD8^+^ | *Not detected* | 0.45 |

Footnotes: only lineage-defining markers are presented in this table. MAIT: mucosal-associated invariant T-cells. TEMRA: terminally differentiated effectors re-expressing CD45RA. NK: natural killer cells. NKT: natural killer T-cells. Treg: T regulators.

1. Expected values found on an average of 5 randomly chosen files from this dataset.

2. Values are given as a percentage of the total number of events used for analysis (201,600 single CD3^+^ events).

**Supplementary Table 4. Exact p-values and test statistics for marker expression comparisons between stimulation conditions, presented in Supplementary Figure 2.**

| **Population** | **Marker** | **Timepoint** | **Stimulation comparison** | **Kruskal p-value** | **Kruskal-Wallis Chi-Square** | **Degrees of freedom** | **Dunn's p-value** | **Dunn's statistic** |
| --- | --- | --- | --- | --- | --- | --- | --- | --- |
| CD4^+^ | CD7 | T0 | NIL-HBHA | 0.012 | 8.75 | 2 | 0.0095 | -2.95 |
| CD4^+^ | CD7 | T1 | NIL-HBHA | 0.029 | 7.03 | 2 | 0.031 | -2.56 |
| CD4^+^ | CCR7 | T1 | NIL-HBHA | 0.036 | 6.62 | 2 | 0.035 | -2.51 |
| CD8^+^ | CD7 | T0 | NIL-HBHA | 0.031 | 6.93 | 2 | 0.038 | -2.49 |
| Tgd | CD7 | T0 | NIL-HBHA | 0.036 | 6.62 | 2 | 0.042 | -2.45 |
| Tgd | CD152 | T2 | NIL-TB2 | 0.016 | 8.26 | 2 | 0.045 | -2.43 |
| Tgd | CD152 | T2 | TB2-HBHA |  |  |  | 0.033 | 2.54 |
| DN | CD152 | T2 | TB2-HBHA | 0.030 | 6.95 | 2 | 0.030 | 2.57 |

Footnotes: here, independent, non-normal continuous variables were analyzed with the two-sided Kruskal–Wallis test with Dunn’s Kruskal–Wallis Multiple Comparisons post-hoc.

**Supplementary Table 5. Exact p-values and test statistics for cluster abundance comparisons during treatment, presented in Figure 3 and Supplementary Figure 5.**

| **Cluster** | **Stim** | **TP** | **Friedman**  **p-value** | **Friedman Chi-Square** | **DF** | **Post-hoc p-value** | **Post-hoc statistic** | **Adjusted p-value** |
| --- | --- | --- | --- | --- | --- | --- | --- | --- |
| 175 | NIL | T0-T1 | 0.012 | 8.78 | 2 | 0.008 | 16 | 0.31 |
| 62 | NIL | T0-T1 | 0.014 | 8.49 | 2 | 0.008 | 16 | 0.31 |
| 119 | NIL | T0-T1 | 0.025 | 7.35 | 2 | 0.045 | 13 | 0.31 |
| 171 | NIL | T0-T1 | 0.044 | 6.26 | 2 | 0.038 | 12.5 | 0.31 |
| 114 | NIL | T0-T1 | 0.015 | 8.4 | 2 | 0.019 | 15 | 0.31 |
| 98 | NIL | T0-T2 | 0.006 | 10.0 | 2 | 0.049 | 12 | 0.31 |
| 38 | NIL | T0-T2 | 0.004 | 11.2 | 2 | 0.021 | 14.5 | 0.31 |
| 176 | NIL | T0-T2 | 0.028 | 7.12 | 2 | 0.023 | 14 | 0.31 |
| 82 | NIL | T0-T2 | 0.033 | 6.82 | 2 | 0.031 | 14 | 0.31 |
| 48 | NIL | T0-T2 | 0.015 | 8.37 | 2 | 0.032 | 13.5 | 0.31 |
| 102 | NIL | T0-T2 | 0.003 | 11.6 | 2 | 0.019 | 14.5 | 0.31 |
| 164 | TB2 | T0-T1 | 0.015 | 8.41 | 2 | 0.024 | 14 | 0.22 |
| 190 | TB2 | T0-T1 | 0.002 | 12.5 | 2 | 0.013 | 16 | 0.20 |
| 16 | TB2 | T0-T1 | 0.055 | 5.81 | 2 | 0.046 | 13.5 | 0.37 |
| 38 | TB2 | T0-T2 | 0.007 | 9.94 | 2 | 0.025 | 15 | 0.22 |
| 70 | TB2 | T0-T2 | 0.001 | 13.7 | 2 | 0.016 | 14.5 | 0.20 |
| 28 | TB2 | T0-T2 | 0.013 | 8.68 | 2 | 0.012 | 16.5 | 0.20 |
| 37 | TB2 | T0-T2 | 0.007 | 9.81 | 2 | 0.009 | 16.5 | 0.20 |
| 137 | TB2 | T0-T2 | 0.001 | 14.9 | 2 | 0.005 | 18 | 0.20 |
| 74 | TB2 | T0-T2 | 0.012 | 8.82 | 2 | 0.015 | 16.5 | 0.20 |
| 102 | TB2 | T0-T2 | 0.022 | 7.61 | 2 | 0.024 | 15.5 | 0.22 |
| 77 | TB2 | T0-T2 | 0.014 | 8.57 | 2 | 0.015 | 16 | 0.20 |
| 56 | HBHA | T0-T1 | 0.023 | 7.53 | 2 | 0.016 | 14 | 0.16 |
| 27 | HBHA | T0-T1 | 0.046 | 6.15 | 2 | 0.04 | 12.5 | 0.16 |
| 128 | HBHA | T0-T1 | 0.013 | 8.74 | 2 | 0.014 | 14 | 0.20 |
| 69 | HBHA | T0-T2 | 0.041 | 6.37 | 2 | 0.03 | 13 | 0.16 |
| 26 | HBHA | T0-T2 | 0.02 | 7.84 | 2 | 0.018 | 14 | 0.16 |
| 38 | HBHA | T0-T2 | 0.026 | 7.28 | 2 | 0.045 | 12.5 | 0.16 |
| 54 | HBHA | T0-T2 | 0.03 | 7.03 | 2 | 0.019 | 13.5 | 0.16 |
| 172 | HBHA | T0-T2 | 0.044 | 6.26 | 2 | 0.038 | 12.5 | 0.16 |
| 91 | HBHA | T0-T2 | 0.018 | 8.04 | 2 | 0.027 | 12.5 | 0.16 |
| 154 | HBHA | T0-T2 | 0.006 | 10.1 | 2 | 0.002 | 16.5 | 0.087 |
| 50 | HBHA | T0-T2 | 0.039 | 6.49 | 2 | 0.032 | 13 | 0.16 |
| 94 | HBHA | T0-T2 | 0.001 | 14.3 | 2 | 0.004 | 19 | >0.01 |
| 49 | HBHA | T0-T2 | 0.008 | 9.69 | 2 | 0.008 | 15 | 0.15 |
| 160 | HBHA | T0-T2 | 0.041 | 6.37 | 2 | 0.034 | 13 | 0.16 |
| 65 | HBHA | T0-T2 | 0.039 | 6.50 | 2 | 0.04 | 12 | 0.16 |

Footnotes**:** For pairwise comparisons between non-independent observations at T0, T1, and T2: the two-sided Friedman rank sum test was performed followed by the Wilcoxon-Nemenyi-Thompson post-hoc. Only clusters within which significant differences were detected were represented. Adjusted p-values refer to.multiple comparisons correction (Benjamini-Hochberg). These values are given as an indication and were not used in cluster selection for phenotype analysis, in order to minimize type II errors. Instead, for discovery of clusters with different abundances. Abbreviations: Stim: stimulation. TP: timepoint. DF: degree of freedom.

**Supplementary Table 6. Pearson’s correlation effect sizes (*r*) presented in Figure 5 for increased clusters.**

|  | **Clus 98** | **Clus 38** | **Clus 176** | **Clus 37** | **Clus 28** | **Clus 70** | **Clus 69** | **Clus 26** | **Clus 172** | **Clus 91** | **Clus 54** |
| --- | --- | --- | --- | --- | --- | --- | --- | --- | --- | --- | --- |
| **Clus 98** | 1 | 0.228 | -0.203 | 0.643 | 0.045 | 0.803 | 0.353 | -0.102 | -0.239 | -0.132 | 0.032 |
| **Clus 38** | 0.228 | 1 | 0.042 | 0.364 | 0.282 | 0.263 | 0.356 | -0.043 | -0.152 | 0.224 | 0.572 |
| **Clus 176** | -0.203 | 0.042 | 1 | -0.042 | -0.049 | -0.128 | -0.131 | -0.065 | -0.116 | 0.018 | -0.215 |
| **Clus 37** | 0.643 | 0.364 | -0.042 | 1 | 0.261 | 0.444 | 0.114 | -0.245 | -0.183 | 0.135 | -0.056 |
| **Clus 28** | 0.045 | 0.282 | -0.049 | 0.261 | 1 | 0.214 | 0.184 | 0.483 | 0.374 | 0.367 | 0.39 |
| **Clus 70** | 0.803 | 0.263 | -0.128 | 0.444 | 0.214 | 1 | 0.691 | 0.207 | -0.259 | 0.048 | 0.279 |
| **Clus 69** | 0.353 | 0.356 | -0.131 | 0.114 | 0.184 | 0.691 | 1 | 0.561 | -0.154 | 0.068 | 0.647 |
| **Clus 26** | -0.102 | -0.043 | -0.065 | -0.245 | 0.483 | 0.207 | 0.561 | 1 | 0.543 | 0.157 | 0.471 |
| **Clus 172** | -0.239 | -0.152 | -0.116 | -0.183 | 0.374 | -0.259 | -0.154 | 0.543 | 1 | -0.081 | 0.039 |
| **Clus 91** | -0.132 | 0.224 | 0.018 | 0.135 | 0.367 | 0.048 | 0.068 | 0.157 | -0.081 | 1 | 0.155 |
| **Clus 54** | 0.032 | 0.572 | -0.215 | -0.056 | 0.39 | 0.279 | 0.647 | 0.471 | 0.039 | 0.155 | 1 |

Footnotes**:** Values indicate Pearson’s *r.* Correlations were calculated based on each cluster’s abundance (percent of total CD3^+^) in samples from all stimulation conditions at treatment initiation (T0). Clus: clusters.

**Supplementary Table 7. Pearson’s correlation effect sizes (*r*) presented in Figure 5 for decreased clusters.**

|  | **Clus 48** | **Clus 82** | **Clus 102** | **Clus 137** | **Clus 77** | **Clus 74** | **Clus 154** | **Clus 50** | **Clus 94** | **Clus 49** | **Clus 160** | **Clus 65** |
| --- | --- | --- | --- | --- | --- | --- | --- | --- | --- | --- | --- | --- |
| **Clus 48** | 1 | 0.377 | 0.412 | 0.137 | 0.462 | 0.725 | 0.485 | -0.093 | 0.711 | 0.565 | 0.286 | 0.372 |
| **Clus 82** | 0.377 | 1 | -0.006 | 0.189 | 0.064 | 0.48 | 0.456 | 0.318 | 0.615 | 0.424 | 0.114 | 0.686 |
| **Clus 102** | 0.412 | -0.006 | 1 | -0.062 | 0.635 | 0.485 | 0.093 | -0.365 | 0.206 | -0.027 | 0.585 | -0.087 |
| **Clus 137** | 0.137 | 0.189 | -0.062 | 1 | -0.072 | -0.054 | 0.226 | -0.113 | 0.066 | 0.237 | -0.008 | -0.076 |
| **Clus 77** | 0.462 | 0.064 | 0.635 | -0.072 | 1 | 0.614 | 0.189 | -0.239 | 0.352 | 0.076 | 0.6 | -0.027 |
| **Clus 74** | 0.725 | 0.48 | 0.485 | -0.054 | 0.614 | 1 | 0.261 | -0.048 | 0.678 | 0.311 | 0.318 | 0.375 |
| **Clus 154** | 0.485 | 0.456 | 0.093 | 0.226 | 0.189 | 0.261 | 1 | 0.288 | 0.471 | 0.513 | 0.145 | 0.64 |
| **Clus 50** | -0.093 | 0.318 | -0.365 | -0.113 | -0.239 | -0.048 | 0.288 | 1 | 0.124 | 0.263 | -0.199 | 0.673 |
| **Clus 94** | 0.711 | 0.615 | 0.206 | 0.066 | 0.352 | 0.678 | 0.471 | 0.124 | 1 | 0.324 | 0.241 | 0.637 |
| **Clus 49** | 0.565 | 0.424 | -0.027 | 0.237 | 0.076 | 0.311 | 0.513 | 0.263 | 0.324 | 1 | -0.063 | 0.452 |
| **Clus 160** | 0.286 | 0.114 | 0.585 | -0.008 | 0.6 | 0.318 | 0.145 | -0.199 | 0.241 | -0.063 | 1 | -0.017 |
| **Clus 65** | 0.372 | 0.686 | -0.087 | -0.076 | -0.027 | 0.375 | 0.64 | 0.673 | 0.637 | 0.452 | -0.017 | 1 |

Footnotes**:** Values indicate Pearson’s *r.* Correlations were calculated based on each cluster’s abundance (percent of total CD3^+^) in samples from all stimulation conditions at treatment initiation (T0). Clus: clusters.

**Supplementary Table 8. Exact p-values and test statistics for cluster abundance comparisons between fast and slow converters, presented in Figure 6 and Supplementary Figure 6.**

| **Cluster ID** | **TP** | **Stimulation** | **N (fast converters)** | **N (slow converters)** | **U statistic** | **p-val** | **adj.**  **p-val** | **Cluster size (cell number)** |
| --- | --- | --- | --- | --- | --- | --- | --- | --- |
| **T0 - significance threshold set at p < 0.026** | | | | | | | | |
| 117 | T0 | NIL | 12 | 4 | 45.5 | 0.0094 | 0.383 | 56 |
| 132 | T0 | NIL | 12 | 4 | 46 | 0.0079 | 0.383 | 81 |
| 134 | T0 | NIL | 12 | 4 | 45.5 | 0.0099 | 0.383 | 152 |
| 147 | T0 | NIL | 12 | 4 | 43 | 0.023 | 0.383 | 79 |
| 148 | T0 | NIL | 12 | 4 | 43.5 | 0.019 | 0.383 | 143 |
| 186 | T0 | NIL | 12 | 4 | 3.5 | 0.0089 | 0.383 | 38 |
| 48 | T0 | NIL | 12 | 4 | 44 | 0.017 | 0.383 | 86 |
| 62 | T0 | NIL | 12 | 4 | 46 | 0.0087 | 0.383 | 187 |
| 76 | T0 | NIL | 12 | 4 | 43.5 | 0.020 | 0.383 | 164 |
| 88 | T0 | NIL | 12 | 4 | 43.5 | 0.020 | 0.383 | 120 |
| 105 | T0 | TB2 | 14 | 4 | 54 | 0.0061 | 0.374 | 81 |
| 118 | T0 | TB2 | 14 | 4 | 51.5 | 0.012 | 0.374 | 109 |
| 134 | T0 | TB2 | 14 | 4 | 52.5 | 0.010 | 0.374 | 180 |
| 147 | T0 | TB2 | 14 | 4 | 50 | 0.021 | 0.374 | 115 |
| 148 | T0 | TB2 | 14 | 4 | 49.5 | 0.025 | 0.374 | 221 |
| 32 | T0 | TB2 | 14 | 4 | 50 | 0.020 | 0.374 | 73 |
| 74 | T0 | TB2 | 14 | 4 | 49.5 | 0.025 | 0.374 | 307 |
| 75 | T0 | TB2 | 14 | 4 | 54.5 | 0.0053 | 0.374 | 113 |
| 89 | T0 | TB2 | 14 | 4 | 50 | 0.020 | 0.374 | 59 |
| 90 | T0 | TB2 | 14 | 4 | 52 | 0.010 | 0.374 | 62 |
| 102 | T0 | HBHA | 11 | 3 | 32 | 0.018 | 0.374 | 128 |
| 132 | T0 | HBHA | 11 | 3 | 31.5 | 0.021 | 0.415 | 97 |
| 134 | T0 | HBHA | 11 | 3 | 31.5 | 0.023 | 0.415 | 146 |
| 148 | T0 | HBHA | 11 | 3 | 33 | 0.012 | 0.415 | 143 |
| 182 | T0 | HBHA | 11 | 3 | 2 | 0.015 | 0.415 | 172 |
| 183 | T0 | HBHA | 11 | 3 | 0 | 0.010 | 0.415 | 193 |
| 52 | T0 | HBHA | 11 | 3 | 31.5 | 0.023 | 0.415 | 116 |
| 62 | T0 | HBHA | 11 | 3 | 32.5 | 0.015 | 0.415 | 305 |
| 75 | T0 | HBHA | 11 | 3 | 31.5 | 0.021 | 0.415 | 101 |

**Supplementary Table 8 - continued**

| **Cluster ID** | | **TP** | **Stimulation** | | **N (fast converters)** | | **N (slow converters)** | | **U statistic** | **p-value** | **adj.**  **p-val** | **Cluster size (cell number)** |
| --- | --- | --- | --- | --- | --- | --- | --- | --- | --- | --- | --- | --- |
| **T1 - significance threshold set at p < 0.031** | | | | | | | | | | | | |
| 116 | T1 | | NIL | 12 | | 4 | | 43 | | 0.023 | 0.583 | 116 |
| 120 | T1 | | NIL | 12 | | 4 | | 43.5 | | 0.019 | 0.583 | 120 |
| 180 | T1 | | NIL | 12 | | 4 | | 6 | | 0.023 | 0.583 | 180 |
| 75 | T1 | | NIL | 12 | | 4 | | 43.5 | | 0.020 | 0.583 | 75 |
| 11 | T1 | | TB2 | 14 | | 4 | | 7.5 | | 0.031 | 0.583 | 11 |
| 117 | T1 | | TB2 | 14 | | 4 | | 50 | | 0.021 | 0.29 | 117 |
| 119 | T1 | | TB2 | 14 | | 4 | | 49 | | 0.027 | 0.29 | 119 |
| 125 | T1 | | TB2 | 14 | | 4 | | 5.5 | | 0.018 | 0.29 | 125 |
| 132 | T1 | | TB2 | 14 | | 4 | | 49.5 | | 0.025 | 0.29 | 132 |
| 134 | T1 | | TB2 | 14 | | 4 | | 55 | | 0.0047 | 0.29 | 134 |
| 146 | T1 | | TB2 | 14 | | 4 | | 49.5 | | 0.025 | 0.29 | 146 |
| 147 | T1 | | TB2 | 14 | | 4 | | 53.5 | | 0.0075 | 0.29 | 147 |
| 148 | T1 | | TB2 | 14 | | 4 | | 52.5 | | 0.010 | 0.29 | 148 |
| 166 | T1 | | TB2 | 14 | | 4 | | 7.5 | | 0.019 | 0.29 | 166 |
| 171 | T1 | | TB2 | 14 | | 4 | | 6 | | 0.021 | 0.29 | 171 |
| 178 | T1 | | TB2 | 14 | | 4 | | 6 | | 0.020 | 0.29 | 178 |
| 180 | T1 | | TB2 | 14 | | 4 | | 7.5 | | 0.028 | 0.29 | 180 |
| 4 | T1 | | TB2 | 14 | | 4 | | 6 | | 0.021 | 0.29 | 4 |
| 57 | T1 | | TB2 | 14 | | 4 | | 5 | | 0.016 | 0.29 | 57 |
| 62 | T1 | | TB2 | 14 | | 4 | | 51 | | 0.016 | 0.29 | 62 |
| 64 | T1 | | TB2 | 14 | | 4 | | 49.5 | | 0.025 | 0.29 | 64 |
| 76 | T1 | | TB2 | 14 | | 4 | | 51 | | 0.016 | 0.29 | 76 |
| 89 | T1 | | TB2 | 14 | | 4 | | 49.5 | | 0.023 | 0.29 | 89 |
| 117 | T1 | | HBHA | 11 | | 3 | | 33 | | 0.011 | 0.29 | 117 |
| 12 | T1 | | HBHA | 11 | | 3 | | 1.5 | | 0.022 | 0.718 | 12 |
| 147 | T1 | | HBHA | 11 | | 3 | | 31.5 | | 0.021 | 0.718 | 147 |
| 180 | T1 | | HBHA | 11 | | 3 | | 1.5 | | 0.020 | 0.718 | 180 |
| 34 | T1 | | HBHA | 11 | | 3 | | 31 | | 0.028 | 0.718 | 34 |
| 62 | T1 | | HBHA | 11 | | 3 | | 32 | | 0.018 | 0.718 | 62 |

**Supplementary Table 8 - continued**

| **Cluster ID** | **TP** | **Stimulation** | **N (fast converters)** | **N (slow converters)** | **U statistic** | **p-val** | **adj.**  **p-val** | **Cluster size (cell number)** |
| --- | --- | --- | --- | --- | --- | --- | --- | --- |
| **T2 - significance threshold set at p < 0.013** | | | | | | | | |
| 112 | T2 | NIL | 12 | 4 | 2 | 0.0073 | 0.145 | 139 |
| 12 | T2 | NIL | 12 | 4 | 2 | 0.0052 | 0.145 | 50 |
| 134 | T2 | NIL | 12 | 4 | 46.5 | 0.0074 | 0.145 | 254 |
| 14 | T2 | NIL | 12 | 4 | 2 | 0.0073 | 0.145 | 107 |
| 169 | T2 | NIL | 12 | 4 | 0 | 0.0031 | 0.145 | 80 |
| 181 | T2 | NIL | 12 | 4 | 3 | 0.012 | 0.145 | 136 |
| 182 | T2 | NIL | 12 | 4 | 0.5 | 0.0021 | 0.145 | 158 |
| 183 | T2 | NIL | 12 | 4 | 1 | 0.0049 | 0.145 | 69 |
| 60 | T2 | NIL | 12 | 4 | 45 | 0.012 | 0.145 | 286 |
| 62 | T2 | NIL | 12 | 4 | 47 | 0.0063 | 0.156 | 115 |
| 66 | T2 | NIL | 12 | 4 | 45.5 | 0.010 | 0.156 | 206 |
| 67 | T2 | NIL | 12 | 4 | 45 | 0.012 | 0.145 | 204 |
| 74 | T2 | NIL | 12 | 4 | 47 | 0.0059 | 0.156 | 128 |
| 81 | T2 | NIL | 12 | 4 | 45 | 0.012 | 0.159 | 206 |
| 112 | T2 | TB2 | 14 | 4 | 4 | 0.012 | 0.159 | 85 |
| 12 | T2 | TB2 | 14 | 4 | 3 | 0.0085 | 0.159 | 269 |
| 134 | T2 | TB2 | 14 | 4 | 52 | 0.012 | 0.159 | 198 |
| 148 | T2 | TB2 | 14 | 4 | 53 | 0.0088 | 0.159 | 117 |
| 166 | T2 | TB2 | 14 | 4 | 2 | 0.0045 | 0.159 | 187 |
| 169 | T2 | TB2 | 14 | 4 | 4 | 0.011 | 0.159 | 263 |
| 180 | T2 | TB2 | 14 | 4 | 2 | 0.0062 | 0.159 | 303 |
| 181 | T2 | TB2 | 14 | 4 | 1.5 | 0.0055 | 0.159 | 217 |
| 182 | T2 | TB2 | 14 | 4 | 1 | 0.0035 | 0.159 | 249 |
| 183 | T2 | TB2 | 14 | 4 | 3 | 0.0064 | 0.159 | 85 |
| 62 | T2 | TB2 | 14 | 4 | 55 | 0.0047 | 0.159 | 342 |
| 88 | T2 | TB2 | 14 | 4 | 52 | 0.012 | 0.159 | 111 |
| 94 | T2 | TB2 | 14 | 4 | 54 | 0.0059 | 0.159 | 65 |
| 98 | T2 | TB2 | 14 | 4 | 3.5 | 0.010 | 0.159 | 206 |
| 183 | T2 | HBHA | 11 | 3 | 0 | 0.011 | 0.293 | 216 |

Footnotes**:** For comparisons between non-normal, independent continuous variables at T0, T1, and T2 separately, the two-sided Mann-Whitney U test was performed. P-values adjusted for multiple comparisons (Benjamini-Hochberg) are given as an indication and were not used in cluster selection for phenotype analysis, in order to minimize type II errors. Instead, for discovery of clusters with different abundances, all p-values were computed for each timepoint, and the p-value corresponding to the null hypothesis being rejected in 5% of all comparisons was used as the significance threshold instead of 0.05. This novel significance threshold enabled to control false discoveries while maintaining an exploratory approach. Abbreviations: TP: timepoint. p-val: p-value. Adj p-val: adjusted P-value. Cluster size refers to the overall number of events per cluster.

**Supplementary Table 9. Exact p-values and test statistics for comparison of PCA scores between fast and slow converters, presented in Figure 7.**

| **Timepoint** | **N(fast converters)** | **N(slow converters)** | **U statistic** | **p-value** |
| --- | --- | --- | --- | --- |
| **Figure 7 - within selected clusters** | | | | |
| **Component 1** | | | | |
| T0 | 25 | 7 | 34 | 0.013 |
| T1 | 25 | 7 | 28 | 0.0051 |
| T2 | 25 | 7 | 25 | 0.0029 |
| **Component 2** | | | | |
| T0 | 25 | 7 | 130 | 0.0542 |
| T1 | 25 | 7 | 72 | 0.503 |
| T2 | 25 | 7 | 146 | 0.00602 |

Footnotes: For comparisons between non-normal, independent continuous variables at T0, T1, and T2 separately, the two-sided Mann-Whitney U test was performed.
